# ﻿Four new species of Russulasubsect.Cyanoxanthinae from China (Russulales, Russulaceae)

**DOI:** 10.3897/mycokeys.107.123304

**Published:** 2024-07-11

**Authors:** Yanliu Chen, Bin Chen, Ruoxi Liang, Shengkun Wang, Mengya An, Jinhua Zhang, Jingying Liang, Yaxin Wang, Xuelian Gao, Junfeng Liang

**Affiliations:** 1 Research Institute of Tropical Forestry, Chinese Academy of Forestry, Guangzhou 510520, China Research Institute of Tropical Forestry, Chinese Academy of Forestry Guangzhou China; 2 Institute of Biological and Medical Engineering, Guangdong Academy of Sciences, Guangzhou 510316, China Institute of Biological and Medical Engineering, Guangdong Academy of Sciences Guangzhou China; 3 Honors College, Northwestern Polytechnical University, Xi’an, 710129, China Northwestern Polytechnical University Xi’an China; 4 Longyandong Forest Farm of Guangdong Province, Guangzhou 510520, China Longyandong Forest Farm of Guangdong Province Guangzhou China

**Keywords:** Ectomycorrhiza, edible fungi, morphology, new species, phylogeny

## Abstract

Four new species of Russulasubsect.Cyanoxanthinae, viz. *Russulaatrochermesina* Y.L. Chen & J.F. Liang, *R.lavandula* Y.L. Chen, B. Chen & J.F. Liang, *R.lilaceofusca* Y.L. Chen & J.F. Liang and *R.perviridis* Y.L. Chen, B. Chen & J.F. Liang, from China are proposed, based on morphological and molecular evidence. *Russulaatrochermesina* can be distinguished by its violet pileus with tuberculate-striate margin, distant lamellae that stain greyish-yellow when bruised, basidiospores ornamented by isolated warts, wide hymenial cystidia on lamellae edges, cystidia content negative reaction in sulphovanillin and branched subterminal cells in pileipellis. *Russulalavandula* has a purplish-white to violet red pileus with a yellow centre, frequently present lamellulae and furcations, stipe often with pale yellow near the base, isolated basidiospores ornamentation and unbranched cuticular hyphal terminations, while *R.lilaceofusca* is characterised by its lilac brown to dark brown pileus, crowded lamellae with lamellulae and furcations, stipe often turning reddish-yellow when bruised, subreticulate basidiospores ornamentation and clavate hymenial cystidia often with capitate appendage whose contents that change to reddish-black in sulphovanillin. *Russulaperviridis* is characterised by its large basidiomata, smooth pileus surface, frequently present lamellulae and furcations, stipe with yellow-brown tinge, globose to broadly ellipsoid basidiospores with subreticulate ornamentation, long hymenial cystidia that turn greyish-black in sulphovanillin and symbiotic with *Quercussemecarpifolia*. Phylogenetic analyses, based on multi-gene ITS+LSU+mtSSU+*rpb2*, indicate that *R.atrochermesina*, *R.lavandula*, *R.lilaceofusca* and *R.perviridis* are closely related to *R.pallidirosea* and *R.purpureorosea*, *R.banwatchanensis*, *R.lakhanpalii* and *R.nigrovirens*, respectively.

## ﻿Introduction

*Russula* Per. is amongst the most ecologically and economically important groups of macrofungi (Looney et al. 2018; Caboň et al. 2019). More than 800 valid species in the genus have been described, but this figure is far below the 2,000 estimated species number (Adamčík et al. 2019). *Russula* is currently divided into nine subgenera, viz. Russulasubg.Archaeae Buyck and V. Hofst., R.subg.Brevipedum Buyck & V. Hofst., R.subg.Compactae (Fr.) Bon, R.subg.Crassotunicatae Buyck & V. Hofst., R.subg.Cremeoochraceae Buyck & X.H. Wang, R.subg.Glutinosae Buyck & X.H. Wang, R.subg.Heterophyllidiae Romagn., R.subg.Malodorae Buyck & V. Hofst. and R.subg.Russula Pers. (Buyck et al. 2018; Buyck et al. 2020; Buyck et al. 2024). The subsect. Cyanoxanthinae Singer belongs to R.subg.Heterophyllidiae and is characterised by the following set of characters: medium to large basidiomata, lamellulae and furcations mostly present, mild to acrid context taste, white to cream spore print, basidiospores with inamyloid suprahilar spots, metachromatic pileipellis in cresyl blue and mainly one-celled pileocystidia. In the wild mushroom markets in south-western China, mushrooms known locally as “Mitangjun” have always been named as *R.cyanoxantha* (Schaeff.) Fr. However, they comprise several species that differ from *R.cyanoxantha*. The species diversity in this subsection has remained unresolved for a long time on the Asian continent, including in China. In the past decade, the number of new species described within this subsection from East and Southeast Asia has increased due to the use of DNA sequence information (Zhao et al. 2015; Zhang et al. 2017; Song et al. 2019; Ghosh et al. 2020; Khatua et al. 2021; Crous et al. 2022; Song 2022).

We aim to clarify the species diversity and geographical distribution of the genus *Russula* in China. From 2012 to 2023, several large-scale fungal surveys were conducted in different regions of China and some interesting specimens were collected. We conducted ITS analyses and found that they belong to the Cyanoxanthinae and are distinct from described species. Subsequently, through morphological comparisons and further multi-gene tree analyses, it was confirmed that they are new species. Here, we propose four new species and provide their morphological descriptions, related illustrations and molecular phylogenetic positions.

## ﻿Materials and methods

### ﻿Sampling and morphological studies

The specimens studied in this paper were collected and photographed from five subtropical provinces in China during 2012–2023. Samples were dried at 50 °C and deposited in the Herbarium of the Research Institute of Tropical Forestry, Chinese Academy of Forestry (RITF).

Macro-morphological features were described using detailed field notes and photographs of fresh basidiomata. Colour designations followed those in the Methuen Handbook of Color (Kornerup and Wanscher 1981). The description of microscopic morphological features followed the template of Adamčík et al. (2019). All micromorphological features were observed using a ZEISS Imager M2 microscope (Carl Zeiss AG, Oberkochen, Germany) with oil immersion lenses at a magnification of 1000×. Basidiospores were observed and measured in Melzer’s Reagent in side view excluding ornamentation. Other microscopic structures were pretreated in 5% potassium hydroxide (KOH) and then stained with 1% Congo red solution. The pileipellis was examined in cresyl blue for the presence of ortho- or metachromatic reactions (Buyck 1989). Sulphovanillin (SV) was used to determine variation in the cystidia content (Caboň et al. 2017). Measurements of basidiospores are expressed as (Min–)AV-SD–AV–AV+SD(–Max), where Min = the minimum value, Max = the maximum value, AV = average, SD = standard deviation and Q stands for the length/width ratio of the basidiospores. A scanning electron microscope (SEM. JEOL JSM-SU8020) was used to illustrate the structure and ornamentation of the basidiospores.

### ﻿DNA extraction, amplified and sequenced

A rapid extraction kit for fungal DNA (Aidlab Biotechnologies Co., Ltd., Beijing, China) was used to extract total DNA from dried specimens. A total of four nuclear loci including ITS (internal transcribed spacer region of ribosomal DNA), LSU (ribosomal nuclear large subunit), mtSSU (ribosomal mitochondrial small subunit) and *rpb2* (second largest subunit of RNA polymerase II) were amplified and sequenced. The ITS regions were amplified with the ITS1/ITS4 primer pairs (White et al. 1990). The LSU regions were amplified with the LR0R/LR5 primer pairs (Vilgalys and Hester 1990). The *rpb2* regions were amplified with the fRPB2-6F/fRPB2-7cR primer pairs (Matheny 2005). The mtSSU regions were amplified with the MS1/MS2 primer pairs (White et al. 1990). Amplifications were performed in 25 µl reactions containing 1.5 µl of Taq DNA polymerase (5U/µl, Thermostable DNA polymerase, BBI), 5 µl of 5× PCR Buffer, 2.5 µl of dNTP (200 µmol/l), 3.5 µl MgCl_2_, 1 µl of each primer (5 µmol/l), 2 µl of DNA template and 8.5 µl of ddH_2_O.

The thermal cycling conditions for ITS, LSU, *rpb2* and mtSSU included an initial denaturation at 95 °C for 5 min, followed by 35 cycles of 95 °C for 30 s (denaturation); 53 °C for 30 s (ITS), 55 °C for 45 s (LSU), 54 °C for 45 s (mtSSU), 56 °C for 1 min (*rpb2*) (annealing), 72 °C for 1 min (elongation) and a final extension at 72 °C for 10 min. PCR products were purified using Bioteke’s Purification Kit (Bioteke Corporation, Beijing, China) and sequenced with an ABI 3730 DNA analyser using an ABI BigDye 3.1 terminator cycle sequencing kit (Shanghai Sangon Biological Engineering Technology and Services Co., Ltd., Shanghai, China). The newly-generated sequences have been submitted to the GenBank database. All sequences used in this study are shown in Table 1.

**Table 1.** GenBank accession numbers for sequences used in this study. The newly-generated sequences are labelled in bold; holotypes are labelled by letter T in parentheses.

Taxon	Voucher	Location	NCBI. No				References
			ITS	LSU	*rpb2*	mtSSU	
***R.atrochermesina***	**RITF6878 (T)**	**Yunnan, south-western China**	**OR907106**	**OR907057**	**OR914538**	**OR934536**	**This study**
***R.atrochermesina***	**RITF6460**	**Yunnan, south-western China**	**OR907107**	**OR907056**		**OR934535**	**This study**
*R.banwatchanensis*	BBH 49228 (T)	Thailand	MT940813	MT940823	MT965687		Crous et al. (2022)
*R.cyanoxantha*	UE29.09.2002-2	France	DQ422033	DQ422033	DQ421970		Buyck et al. (2008)
*R.cyanoxantha*	FH 12–201	Germany	KR364093	KR364225	KR364341		De Crop et al. (2017)
*R.dinghuensis*	K15052704 (T)	Guangdong, southern China	KU863581	MK881922		MK882050	Zhang et al. (2017)
*R.dinghuensis*	RITF6860	Guangdong, southern China	**OR907119**	**OR907061**	**OR914549**	**OR934507**	**This study**
*R.dinghuensis*	RITF5885	Guangdong, southern China	**OR907120**		**OR914550**	**OR934503**	**This study**
*R.dinghuensis*	RITF5142	Jiangxi, central China	**OR907123**	**OR907058**	**OR914551**	**OR934502**	**This study**
*R.dinghuensis*	RITF6855	Guangxi, southern China	**OR907121**	**OR907060**	**OR914552**	**OR934504**	**This study**
*R.dinghuensis*	RITF6740	Guangxi, southern China	**OR907122**	**OR907059**	**OR914553**	**OR934506**	**This study**
*R.dinghuensis*	RITF6859	Guangdong, southern China	**OR907124**	**OR907062**	**OR914554**	**OR934505**	**This study**
R.flavobrunneavar.violaceotincta	71/BB 06.050	Madagascar		KU237468	KU237754	KU237312	Buyck et al. (2018)
*R.fusiformata*	K15052703 (T)	Guangdong, southern China	MK049978	MK881942		MK882070	Song (2022)
*R.fusiformata*	RITF6671	Zhejiang, eastern China	**OR907108**	**OR907049**	**OR914567**	**OR934501**	**This study**
*R.fusiformata*	RITF6487	Jiangxi, central China	**OR907109**		**OR914563**	**OR934500**	**This study**
*R.lakhanpalii*	CAL 1795 (T)	India	NR_173867				
*R.lakhanpalii*	RITF2600	Hubei, central China	**OR907090**	**OR907076**		**OR934525**	**This study**
*R.lakhanpalii*	RITF6973	Yunnan, south-western China	**OR907089**		**OR914542**	**OR934526**	**This study**
*R.lakhanpalii*	RITF6868	Yunnan, south-western China	**OR907091**	**OR907078**	**OR914543**	**OR934528**	**This study**
*R.lakhanpalii*	RITF6474	Yunnan, south-western China	**OR907092**	**OR907077**	**OR914544**	**OR934527**	**This study**
*R.lakhanpalii*	RITF6940	Yunnan, south-western China	**OR907088**	**OR907079**		**OR934529**	**This study**
*R.langei*	450/BB 07.792	France		KU237510	KU237796	KU237356	Buyck et al. (2018)
***R.lavandula***	**RITF6329**	**Yunnan, south-western China**	**OR907083**	**OR907065**	**OR914534**	**OR934513**	**This study**
***R.lavandula***	**RITF6340**	**Yunnan, south-western China**	**OR907085**	**OR907066**	**OR914535**	**OR934515**	**This study**
***R.lavandula***	**RITF3196**	**Yunnan, south-western China**	**OR907086**	**OR907067**		**OR934512**	**This study**
***R.lavandula***	**RITF3282 (T)**	**Yunnan, southwestern China**	**OR907087**	**OR907068**	**OR914537**	**OR934511**	**This study**
***R.lavandula***	**RITF6349**	**Yunnan, south-western China**	**OR907084**	**OR907069**	**OR914536**	**OR934514**	**This study**
***R.lilaceofusca***	**RITF6330 (T)**	**Yunnan, south-western China**	**OR907102**	**OR907075**	**OR914539**	**OR934530**	**This study**
***R.lilaceofusca***	**RITF2645**	**Hubei, central China**	**OR907093**	**OR907074**	**OR914540**	**OR934531**	**This study**
***R.lilaceofusca***	**RITF2631**	**Hubei, central China**			**OR914541**		**This study**
***R.lilaceofusca***	**RITF3761**	**Guizhou, south-western China**	**OR907094**				**This study**
*R.lilacina*	MMCR00191	Thailand	MT940809	MT940819	MT965685		Crous et al. (2022)
*R.lotus*	HKAS79209 (T)	Guangdong, southern China	MG214688	MG214695			Li and Deng (2018)
*R.lotus*	**RITF3330**	**Yunnan, south-western China**		**OR907050**			**This study**
*R.maguanensis*	HKAS 102277 (T)	Yunnan, south-western China	MH724918	MH714537	MH939989		Wang et al. (2019)
*R.nigrovirens*	HKAS 55222 (T)	Yunnan, south-western China	KP171173				Zhao et al. (2015)
*R.nigrovirens*	RITF6408	Yunnan, south-western China	**OR907095**	**OR907073**	**OR914568**	**OR934521**	**This study**
*R.pallidirosea*	UTC 00274382 (T)	USA	NR_153259				Kropp (2016)
***R.perviridis***	**RITF3131 (T)**	**Yunnan, south-western China**	**OR907098**	**OR907072**	**OR914548**	**OR934523**	**This study**
***R.perviridis***	**RITF2912**	**Xizang, western China**	**OR907100**	**OR907070**	**OR914547**	**OR934522**	**This study**
***R.perviridis***	**RITF6983**	**Yunnan, south-western China**	**OR907099**				**This study**
***R.perviridis***	**RITF6982**	**Yunnan, south-western China**	**OR907101**	**OR907071**	**OR914545**	**OR934524**	**This study**
***R.perviridis***	**RITF6734**	**Sichuan, south-western China**			**OR914546**		**This study**
***R.perviridis***	**RITF3143**	**Yunnan, south-western China**	**OR907097**				**This study**
***R.perviridis***	**RITF6790**	**Xizang, western China**	**OR907096**				**This study**
*R.phloginea*	CNX530524068 (T)	Yunnan, south-western China	MK860701	MK860704		MK860708	Song et al. (2019)
*R.phloginea*	RITF6904	Yunnan, south-western China	**OR907113**				**This study**
*R.phloginea*	RITF6905	Yunnan, south-western China	**OR907116**	**OR907051**	**OR914555**	**OR934516**	**This study**
*R.phloginea*	RITF6906	Yunnan, south-western China	**OR907117**	**OR907055**	**OR914558**	**OR934520**	**This study**
*R.phloginea*	RITF6907	Yunnan, south-western China	**OR907114**	**OR907052**	**OR914556**	**OR934517**	**This study**
*R.phloginea*	RITF6908	Yunnan, south-western China	**OR907115**	**OR907054**	**OR914557**	**OR934518**	**This study**
*R.phloginea*	RITF6914	Yunnan, south-western China	**OR907118**	**OR907053**	**OR914559**	**OR934519**	**This study**
*R.pseudocyanoxantha*	CUH AM177 (T)	India	NR_173166				Khatua et al. (2021)
*R.purpureorosea*	H17050506 (T)	Guangdong, southern China	MK049976	MK881941		MK882069	Song (2022)
*R.purpureorosea*	RITF6835	Guangdong, southern China	**OR907104**	**OR907082**	**OR914565**	**OR934533**	**This study**
*R.purpureorosea*	RITF6834	Guangdong, southern China	**OR907103**	**OR907081**	**OR914566**	**OR934534**	**This study**
*R.purpureorosea*	RITF5886	Guangdong, southern China	**OR907105**	**OR907080**	**OR914564**	**OR934532**	**This study**
*R.purpureoviridis*	BBH 49226 (T)	Thailand		MT940817	MT965684		Crous et al. (2022)
*R.subpallidirosea*	K15052818 (T)	Guangdong, southern China	KU863582	MK881923		MK882051	Zhang et al. (2017)
*R.subpallidirosea*	RITF3219	Yunnan, south-western China	**OR907111**	**OR907063**	**OR914560**	**OR934508**	**This study**
*R.subpallidirosea*	RITF3343	Yunnan, south-western China	**OR907112**		**OR914562**	**OR934509**	**This study**
*R.subpallidirosea*	RITF6264	Yunnan, south-western China	**OR907110**	**OR907064**	**OR914561**	**OR934510**	**This study**
*R.substriata*	HKAS 102278 (T)	Yunnan, south-western China	MH724921	MH714540	MH939992		Wang et al. (2019)
*R.variata*	BPL241	USA	KT933959	KT933818	KT933889		Looney et al. (2016)

### ﻿DNA sequence alignments and molecular phylogenetic analyses

Representative sequences of species belonging to the Cyanoxanthinae were obtained from the GenBank database, based on recent published studies (Crous et al. 2022; Song 2022). Two species of subsect. Substriatinae, *R.maguanensis* J. Wang, X.H. Wang, Buyck & T. Bau and *R.substriata* J. Wang, X.H. Wang, Buyck & T. Bau, were used as outgroups. Sequences of four genes were aligned separately using MAFFT 7.0 (http://mafft.cbrc.jp/alignment/server/) and the alignment was manually refined using BioEdit v.7.0.9 (Hall 1999).

Phylogenetic analyses, based on a multi-gene matrix, were conducted using both Randomised Accelerated Maximum Likelihood (RAxML) and Bayesian Inference (BI). A partition homogeneity test (PHT) was performed using heuristic searches with PAUP* 4.0a (Swofford 2003) to evaluate incongruence amongst individual genes. The sequences were then concatenated using Phyutility v.2.2 (Smith and Dunn 2008) for multi-gene analyses as no supported conflicts (P < 0.5). The final aligned dataset was submitted to TreeBASE (31001). A mixed-model (partitioned) scheme was used for ML and BI analyses, ITS, LSU, mtSSU and *rpb2*. The best-fit model of sequence evolution for each gene was estimated using MrModelTest 2.3 (Nylander 2004), based on the Akaike Information Criterion. The best model for the BI analysis of the ITS and LSU datasets was the GTR+I+G model; the SYM+G model was optimal for *rpb2*; and the GTR+I model was optimal for mtSSU. RaxML 7.0.3 (Stamatakis 2006) and MrBayes v.3.2 (Ronquist et al. 2012) were used to perform the RAxML and BI analyses, respectively. Maximum Likelihood analysis was conducted using non-parametric bootstrapping with 1,000 replicates. Bootstrap support (BS) above 70% (Nuhn et al. 2013) was considered significant. BI analysis was performed using the Metropolis-coupled Markov Chain Monte Carlo method. Four chains were run for 1,500,000 generations and trees were sampled every 100 generations. Other parameters were kept at their default settings. The analysis was terminated when the average standard deviation of the splitting frequency was below 0.01 and the PSRF values for all parameters were near 1. Bayesian posterior probabilities (BPP) were calculated after discarding the first 25% of the samples as burn-in and branches with BPP over 0.95 were considered significantly supported.

## ﻿Results

### ﻿Phylogeny

**Figure 1.**
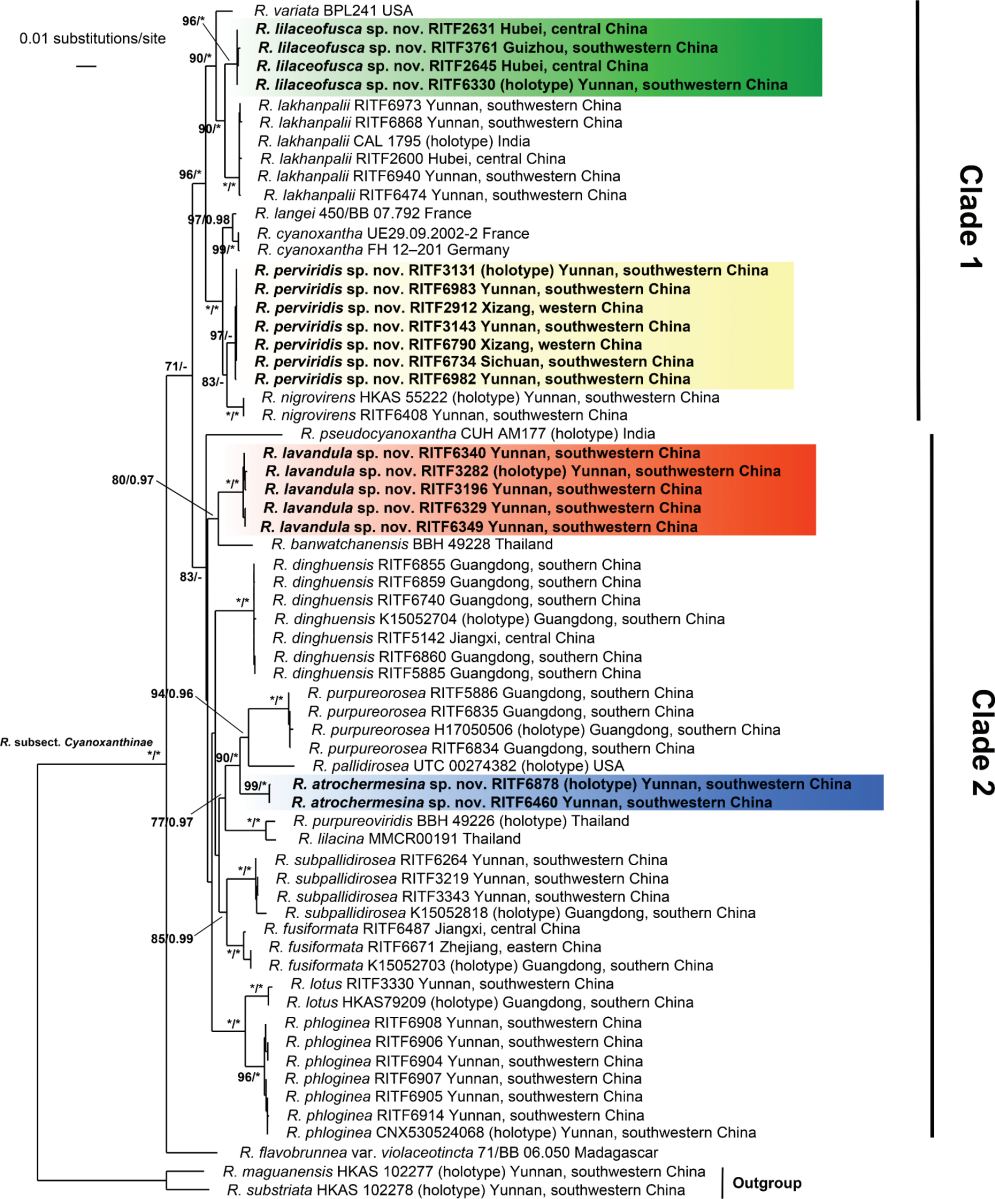
The RAxML likelihood tree based on the four-gene dataset (ITS+LSU+mtSSU+*rpb2*). Support values (BS > 70%, BPP > 0.95) are displayed above or below the branches, with the maximum values of BS (= 100%) and BPP (= 1.0) represented by asterisks (*). The four new species are marked with different colours.

The four-gene dataset (ITS+LSU+mtSSU+*rpb2*) included 65 samples representing 22 species. The matrix contains a total of 2,786 nucleotide sites, including 652 ITS sites, 896 LSU sites, 516 mtSSU sites and 722 *rpb2* sites. The tree topologies of both the RAxML and BI analyses were similar and only the tree inferred by the ML analysis is displayed, but with both BS and BPP values (Fig. 1).

Our molecular phylogenetic analyses show that *Cyanoxanthinae* can be divided into two major supported lineages and one species from Madagascar. All samples from China fell into major clades and represent 12 species, of which four are new species. The four new species proposed each formed a well-supported branch and were distinct from other related taxa. *Russulaatrochermesina* Y.L. Chen & J.F. Liang, sp. nov. (BS = 99%, BPP = 1.00) was closely related to *R.pallidirosea* Kropp and *R.purpureorosea* Y. Song. *Russulalavandula* Y.L. Chen, B. Chen & J.F. Liang, sp. nov. (100% BS and 1.00 BPP) was sister to *R.banwatchanensis* Sommai, Pinruan, Somrith. & Luangsa-ard (80% BS and 0.97 BPP). *Russulalilaceofusca* Y.L. Chen & J.F. Liang, sp. nov. (BS = 96%, BPP = 1.00) was allied to *R.lakhanpalii* (90% BS and 1.00 BPP). *Russulaperviridis* Y.L. Chen, B. Chen & J.F. Liang, sp. nov. (BS = 97%, BPP < 0.95) was close to *R.nigrovirens* Q. Zhao, Y.K. Li & J.F. Liang (BS = 83%, BPP < 0.95).

## ﻿Taxonomy

### ﻿ Russula atrochermesina

Taxon classificationFungiRussulalesRussulaceae

Y.L. Chen & J.F. Liang, sp. nov.

95637F13-8C52-586F-8BB7-E303063A60DB

851267

**Figure 2.**
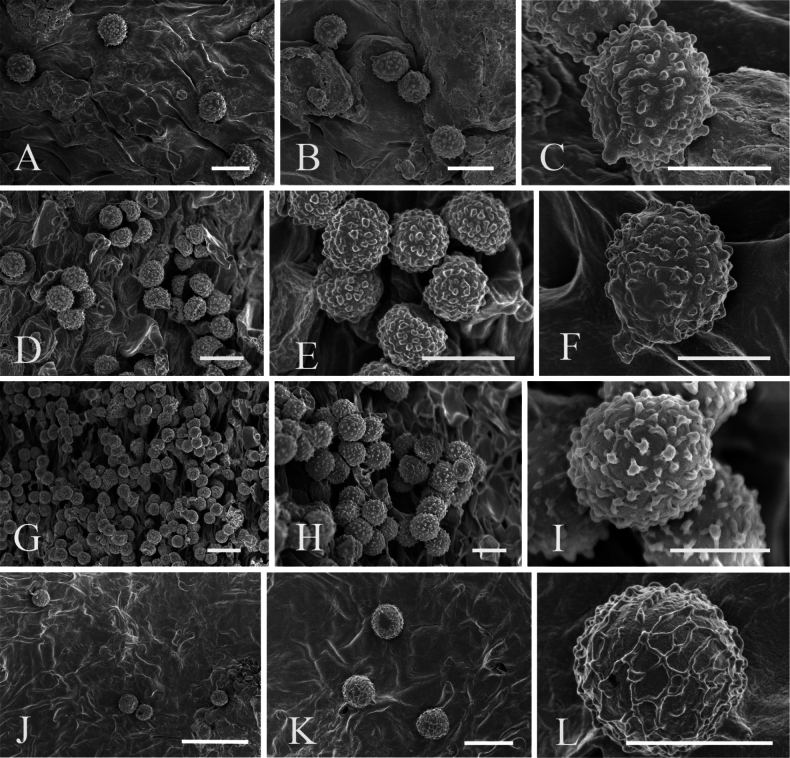
SEM photos of basidiospores of the four new species **A–C***Russulaatrochermesina* (RITF6878, holotype) **D–F***Russulalavandula* (RITF6329) **G–I***Russulalilaceofusca* (RITF6330, holotype) **J–L***Russulaperviridis* (RITF3131, holotype). Scale bars: 20 µm (**G**); 10 µm (**A, B, D, E, H, J, K**); 5 µm (**C, F, I, L**).

**Figure 3.**
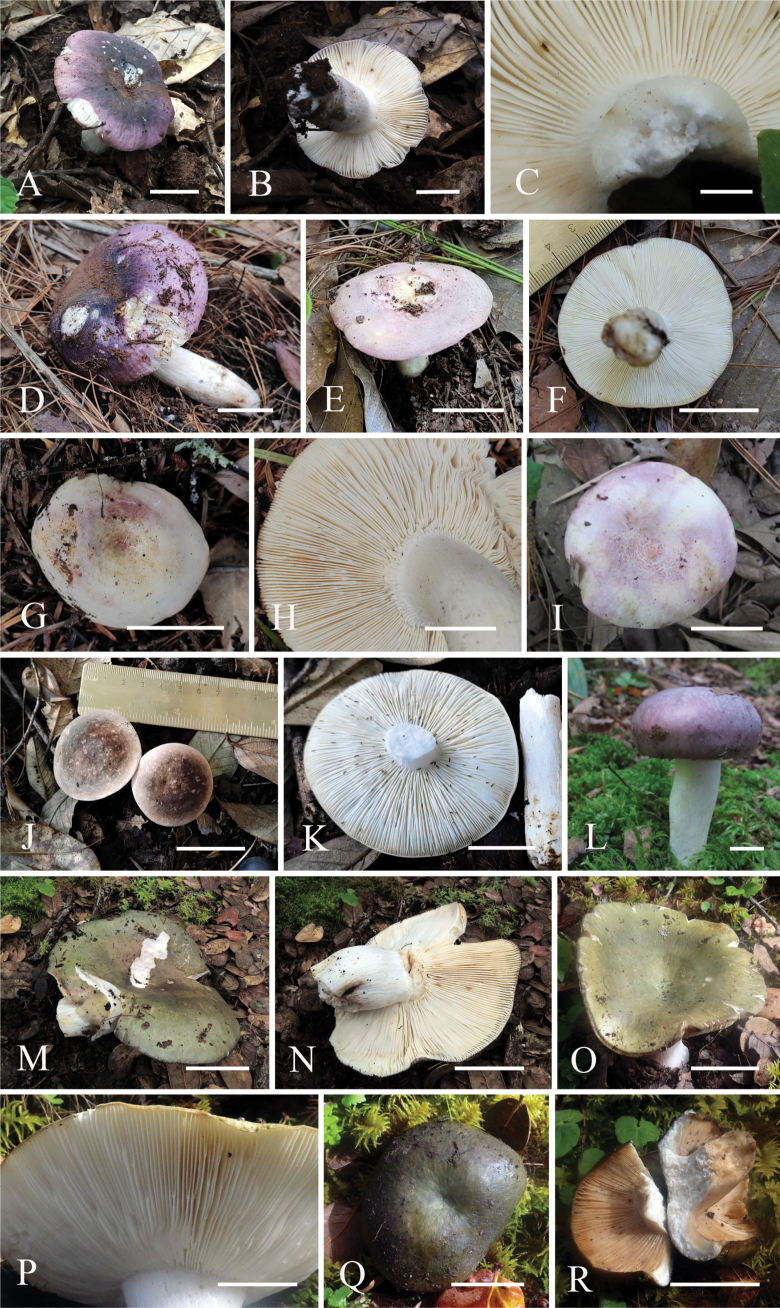
Fruiting bodies photos of four new species **A–D***Russulaatrochermesina* (A-C-RITF6878, holotype, D-RITF6460) **E–I***Russulalavandula* (E-F-RITF6329, G-H-RITF6340, I-RITF3282, holotype) **J–L***Russulalilaceofusca* (J-K-RITF6330, holotype, L-RITF2631) **M–R***Russulaperviridis* (M-N-RITF3131, holotype, O-P-RITF6982, Q-R-RITF6983). Scale bars: 40 mm (**A, B, D–G, I, J, M–O**); 5 mm (**C, L**); 20 mm (**H, K, P, Q, R**).

**Figure 4.**
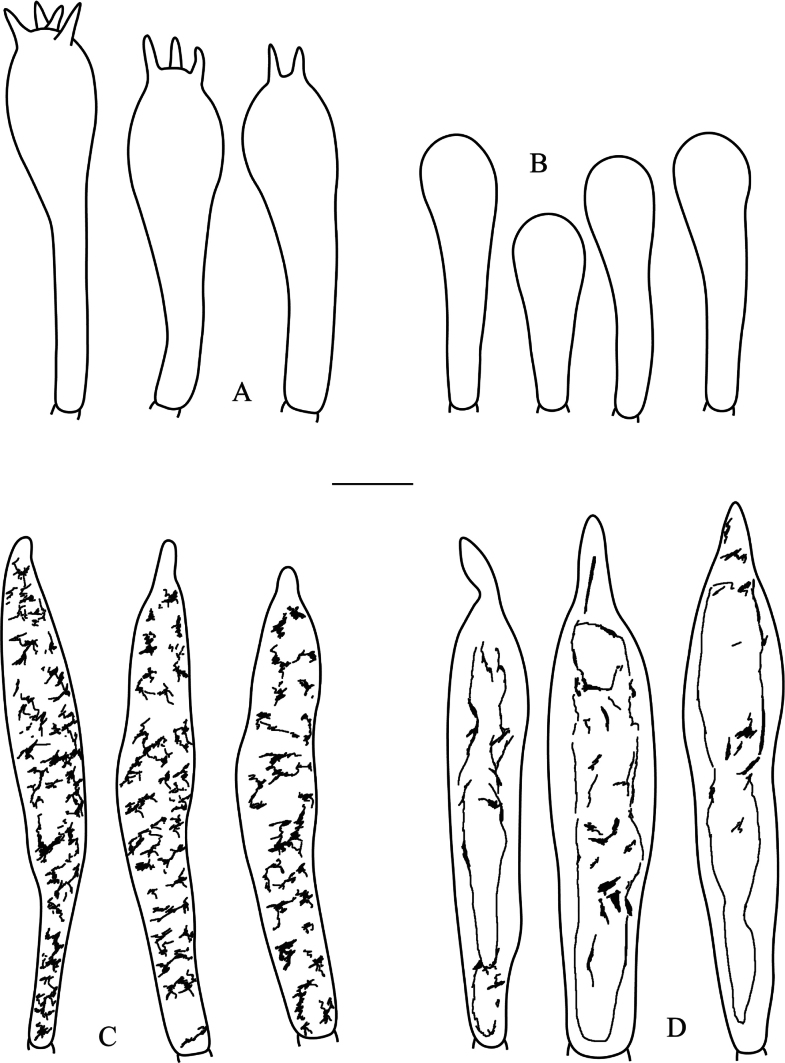
*Russulaatrochermesina* (RITF6878, holotype) **A** basidia **B** basidiola **C** hymenial cystidia on lamellae sides **D** hymenial cystidia on lamellae edges. Scale bar: 10 µm.

**Figure 5.**
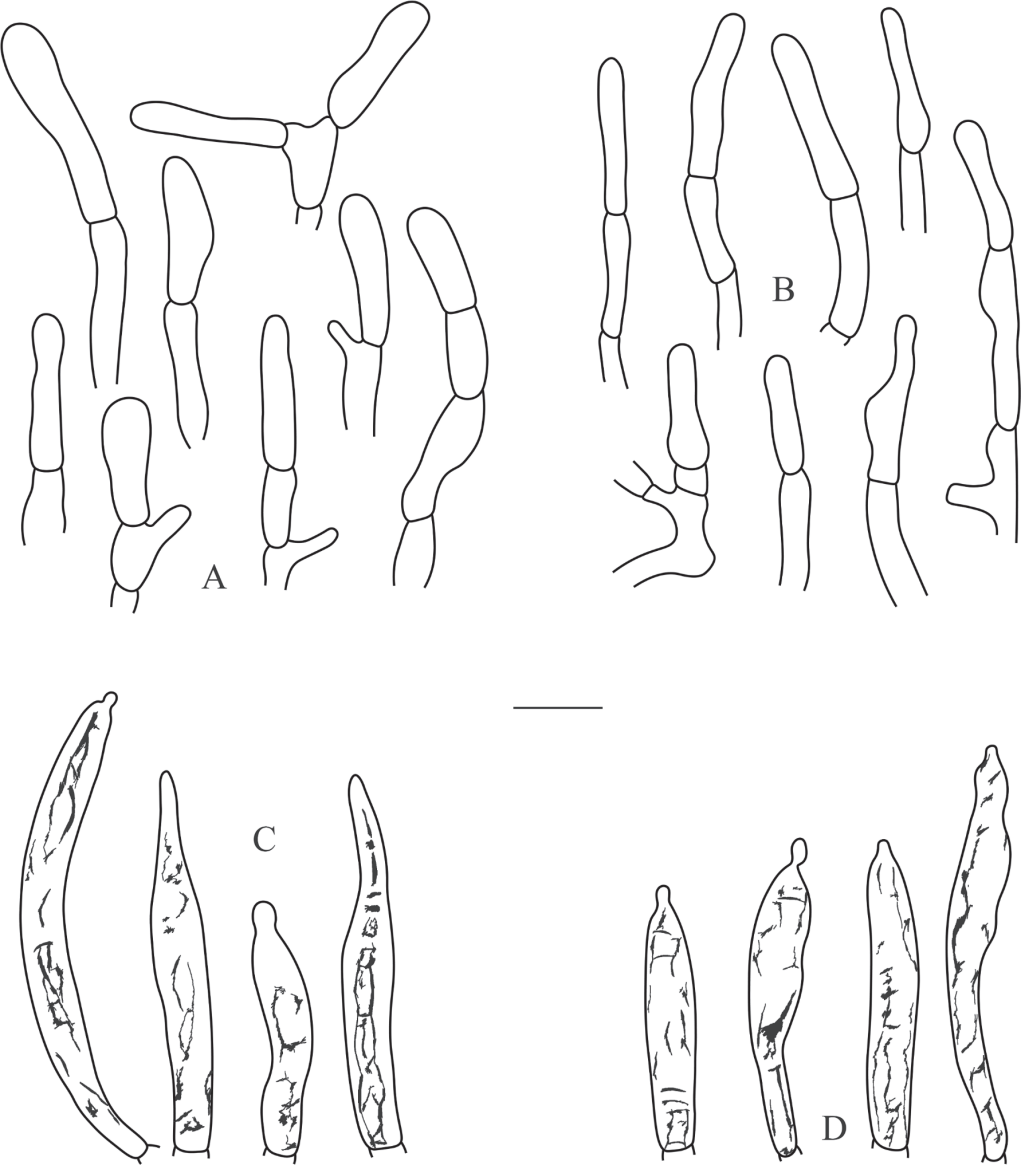
*Russulaatrochermesina* (RITF6878, holotype) **A** hyphal terminations near the pileus margin **B** hyphal terminations near the pileus centre **C** pileocystidia near the pileus margin **D** pileocystidia near the pileus centre. Scale bar: 10 µm.

Figs 2A–C , 3A–D , 4 , 5

#### Diagnosis.

*Russulaatrochermesina* can be distinguished by its violet pileus with tuberculate-striate margin, distant lamellae that stain greyish-yellow when bruised, wide hymenial cystidia on lamellae edges, cystidia content negative reaction in sulphovanillin, branched subterminal cells in pileipellis.

#### Holotype.

China, Yunnan Province, Chuxiong Yi Autonomous Prefecture, Lufeng City, Tuoan Township, 25°14'35"N, 101°46'23"E, alt. 2250 m, 7 Sep 2023, Y.L. Chen (RITF6878).

#### Etymology.

‘*atrochermesina*’ refers to the dark purple colour of pileus.

#### Description.

***Basidiomata*** medium to large-sized; pileus 75–115 mm in diameter, initially hemispherical when young, convex to applanate when mature, margin tuberculate-striate, not cracked; surface dry, glabrous, peeling readily, violet white (16A2) to pale violet (16A3), dark violet (16F6) in the centre. ***Lamellae*** adnate, 5–8 per cm near pileus margin, cream, staining greyish-yellow (2B5) when bruised; lamellulae present and irregular in length; furcations frequently present near the stipe; edge entire and concolorous. ***Stipe*** 80–170 × 25–40 mm, cylindrical to subcylindrical, white (1A1), often with yellowish-white (1A2) tinge, solid. ***Context*** white (1A1), unchanging when bruised, compact, 5 mm thick in half of the pileus radius; taste mild; odour inconspicuous. ***Spore print*** not observed.

***Basidiospores*** (6.3–)6.7–7.2–7.6(–8.7) × (5.3–)5.8–6.2–6.5(–7.4) µm, Q = (1.05–)1.11–1.15–1.20(–1.25), subglobose to broadly ellipsoid, hyaline in 5% KOH; ornamentation of small to medium, dense (5–9 in a 3 μm diam. circle) amyloid warts, less than 0.6 μm high, mostly isolated or fused in pairs, occasionally fused by short lines; suprahilar plage inamyloid. ***Basidia*** (33.0–)37.0–43.0–48.5(–52.0) × (9.0–)10.0–11.5–12.5(–14.0) µm, clavate, 2- to 4-spored, thin-walled; basidiola clavate, ca. 8.0–12.5 µm wide. ***Hymenial cystidia on lamellae sides*** moderately numerous, (52.5–)55.0–60.5–65.5(–69.5) × (7.0–)8.0–8.5–9.5(–10.0) µm, fusiform, apically acute, usually with an appendage, thin-walled; contents heteromorphous, yellow in sulphovanillin. ***Hymenial cystidia on lamellae edges*** longer and wider than those on lamellae sides, (54.5–)58.0–65.0–71.5(–75.5) × (8.0–)9.0–10.5–11.5(–13.0) µm, fusiform, apically mostly acute, usually with an appendage, thin-walled; contents crystalline, yellow in sulphovanillin. ***Marginal cells*** undifferentiated. ***Pileipellis*** hyphae of all tissues metachromatic in cresyl blue, sharply delimited from the underlying context, two-layered, gelatinised; suprapellis 260–400 µm deep, composed of densely arranged and erect hyphal terminations; subpellis 150–200 µm deep, composed of horizontally orientated and 2–9 μm wide hyphae. Hyphal terminations near the pileus margin branched, thin-walled; terminal cells (9.0–)13.5–18.0–22.5(–27.5) × (2.5–)3.5–4.0–5.0 µm, mainly clavate, occasionally cylindrical, apically mostly obtuse; subterminal cells usually shorter, ca. 3.0–5.5 µm wide, branched. Hyphal terminations near the pileus centre slightly narrower than those near the pileus margin; terminal cells (10.0–)13.5–17.5–21.5(–26.5) × (2.5–)3.0–3.5–4.0(–5.0) µm, cylindrical, slightly attenuated. ***Pileocystidia*** near the pileus margin always 1-celled, (22.5–)28.5–36.0–44.0(–50.0) × (4.0–)4.5–5.0–6.0(–6.5) µm, dispersed, clavate, occasionally fusiform, apically usually obtuse, sometimes with a round or elliptical appendage, thin-walled; contents crystalline, no reaction in sulphovanillin. Pileocystidia near the pileus centre slightly narrower than those near the pileus margin, (30.0–)32.0–38.0–44.0(–51.5) × (3.5–)4.0–4.5–5.0(–5.5) µm.

#### Habitat.

On the ground under mixed forests dominated by *Castanopsissclerophylla* and *Pinusyunnanensis*.

#### Known distribution.

South-western China (Yunnan Province).

#### Additional specimens examined.

China, Yunnan Province, Chuxiong Yi Autonomous Prefecture, Lufeng City, Tuoan Township, 25°14'34"N, 101°45'39"E, alt. 2300 m, 28 Jul 2022, X.L. Gao (RITF6460).

#### Notes.

Phylogenetic analyses showed that *R.atrochermesina* is related to *R.pallidirosea*, *R.purpureorosea* and *R.purpureoviridis* Khamsuntorn, Lueangjaroenkit, Sommai & Pinruan. However, *R.pallidirosea* from American Samoa has a pallid to pinkish pileus and smaller hymenial cystidia on the lamellae edges (40.0–55.0 × 5.0–7.0 µm) and is associated with tropical trees (Kropp 2016). *Russulapurpureorosea* differs in the following set of characters: pale pinkish-purple pileus, lamellae furcations absence, narrower hymenial cystidia on lamellae edges (54.0–60.0–95.0 × 7.0–8.5–10.0 µm) and unbranched hyphal terminations in the pileipellis (Song 2022). *Russulapurpureoviridis*, originally described from Thailand, differs in a greyish-green pileus, narrower hymenial cystidia on lamellae edges (45.0–77.5 × 7.5–10.0 µm), constricted terminal cells in the pileipellis and larger pileocystidia (52.5–87.5 × 5.0–8.5 µm) (Crous et al. 2022). The morphology of *R.atrochermesina* is similar to *R.fusiformata*. However, *R.fusiformata* lacks lamellulae and furcations and has narrower hymenial cystidia on lamellae edges (35.5–52.0–78.0 × 5.0–9.0–11.0 µm) and shorter and wider terminal cells near the pileal centre (5.5–14.0 × 3.0–8.0 µm) (Song 2022).

### ﻿ Russula lavandula

Taxon classificationFungiRussulalesRussulaceae

Y.L. Chen, B. Chen & J.F. Liang, sp. nov.

8A0F09C7-131C-5EF8-A92D-E4E99F041CFD

851264

**Figure 6.**
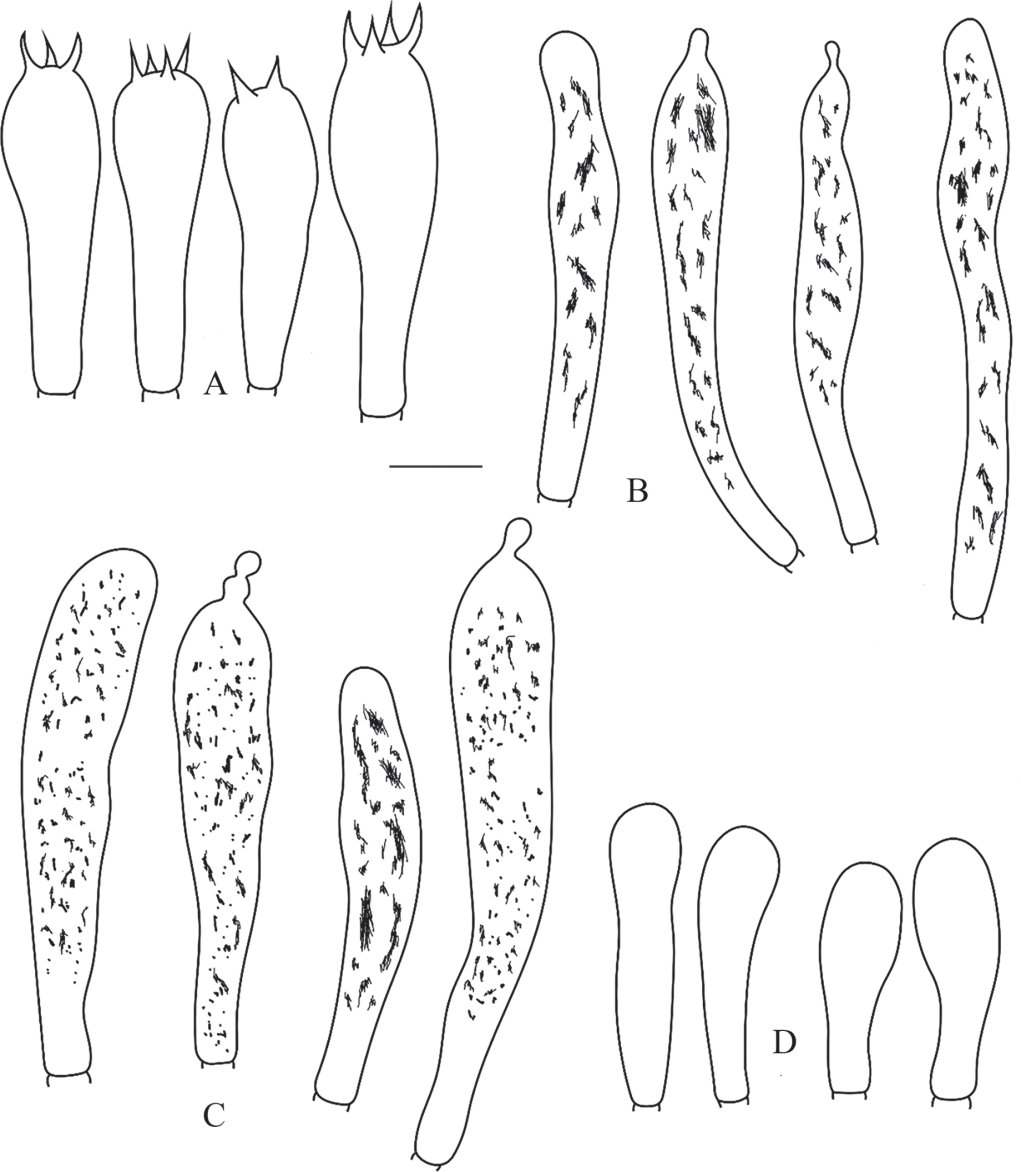
*Russulalavandula* (RITF3282, holotype) **A** basidia **B** hymenial cystidia on lamellae sides **C** hymenial cystidia on lamellae edges **D** basidiola. Scale bar: 10 µm.

**Figure 7.**
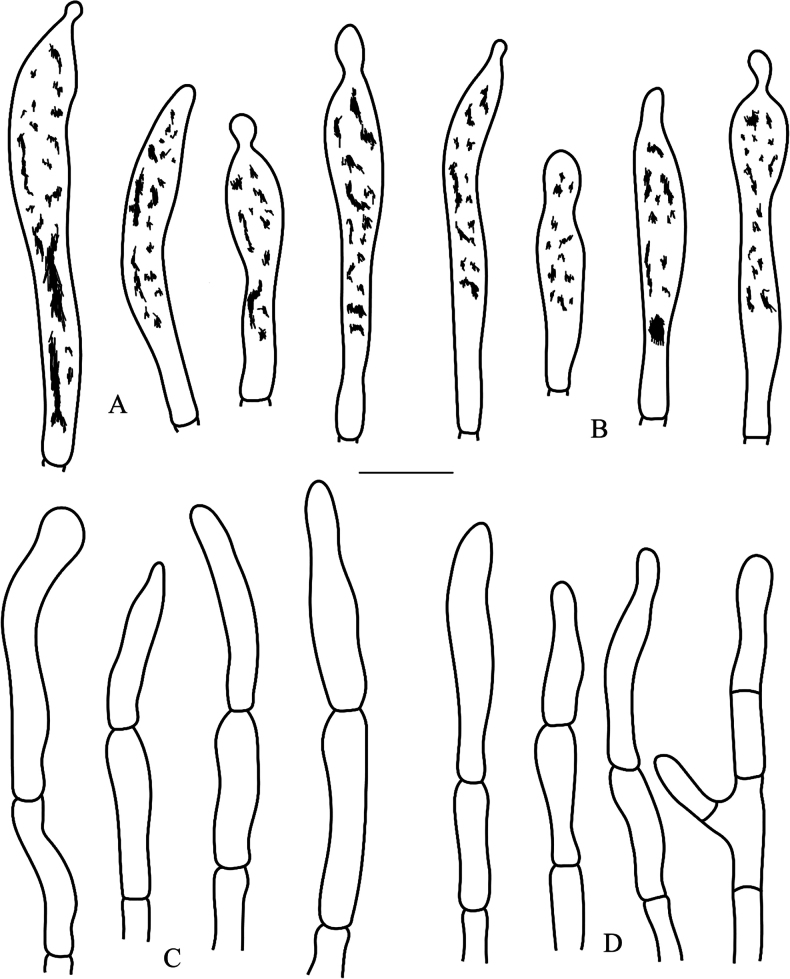
*Russulalavandula* (RITF3282, holotype) **A** pileocystidia near the pileus margin **B** pileocystidia near the pileus centre **C** hyphal terminations near the pileus margin **D** hyphal terminations near the pileus centre. Scale bar: 10 µm.

Figs 2D–F , 3E–I , 6 , 7

#### Diagnosis.

*Russulalavandula* is characterised by its purplish-white to violet red pileus with a yellow centre, frequently present lamellulae and furcations, stipe often with pale yellow near the base, isolated basidiospores ornamentation and unbranched cuticular hyphal terminations. It is mainly distinguished from *R.lotus* Fang Li by its frequently present lamellulae and furcations, subglobose to broadly ellipsoid basidiospores, moderately numerous and narrower hymenial cystidia on lamellae sides and shorter cuticular terminal cells.

#### Etymology.

‘*lavandula*’ refers to the colour of its pileus similar to lavender.

#### Holotype.

China, Yunnan Province, Kunming City, Wild Duck Lake, 25°07'34"N, 102°51'42"E, alt. 2100 m, 27 Jul 2014, H.J. Li (RITF3282).

#### Description.

***Basidiomata*** medium-sized; pileus 40–80 mm in diameter, initially hemispherical when young, convex to applanate with a depressed centre after maturity; margin incurved, striation short or inconspicuous, cracked after maturity; surface dry, glabrous, peeling readily, locally cracking into pale yellow (2A3), purplish-white (14A2) to greyish-magenta (13D5 or 14E6) patches, rose (13B3), purplish-white (14A2) to violet red (14B6), sometimes white (1A1) at the margin, yellowish-white (1A2) to golden yellow (4C7) in the centre. ***Lamellae*** adnate to slightly adnexed, 10–13 per cm near pileus margin, white (1A1), unchanging when bruised, 3–4 mm wide; lamellulae usually present and irregular in length; furcations frequently present throughout the lamellae; edge entire and concolorous. ***Stipe*** 40–60 × 16–25 mm, cylindrical, flexuous and tapering towards the base, white (1A1), often with pale yellow tinge near the base, solid. ***Context*** white (1A1), unchanging when bruised, 3–4 mm thick in half of the pileus radius; taste mild; odour inconspicuous. ***Spore print*** not observed.

***Basidiospores*** (6.5–)7.1–7.7–8.3(–9.2) × (5.9–)6.4–6.9–7.5(–8.0) µm, Q = (1.01–)1.05–1.11–1.17(–1.24), subglobose to broadly ellipsoid, hyaline in 5% KOH; ornamentation of small, moderately distant to dense (6–8 in a 3 μm diam. circle) amyloid warts, less than 0.5 μm high, mostly isolated, occasionally connected by short line connections or ridges, not forming a reticulum; suprahilar plage indistinct, inamyloid. ***Basidia*** (24.0–)28.5–33.5–39.0(–43.5) × (6.0–)7.0–8.5–9.5(–10.5) µm, clavate, 2- to 4-spored, thin-walled; basidiola clavate or subcylindrical, ca. 5–10 µm wide. ***Hymenial cystidia on lamellae sides*** moderately numerous, (42.5–)47.0–54.5–62.5(–64.5) × (5.0–)6.5–7.5–8.5 µm, clavate or fusiform, apically mostly obtuse, partially acute, sometimes with a 4–6 µm and papillate appendage, thin-walled; contents granulose or heteromorphous, reddish-black in sulphovanillin. ***Hymenial cystidia on lamellae edges*** shorter, but wider than those on lamellae sides, (35.0–)39.0–46.0–52.5(–56.0) × (6.0–)7.0–8.1–9.2(–10.0) µm, mostly clavate or subcylindrical, apically mostly obtuse, occasionally with 2–5 µm long, papillate or moniliform appendage. ***Marginal cells*** undifferentiated. ***Pileipellis*** only hyphae of suprapellis metachromatic in cresyl blue, sharply delimited from the underlying context, 300–450 µm deep, two-layered, gelatinised; suprapellis 180–200 µm deep, composed of densely arranged and prostrate to erect hyphal terminations; subpellis 140–260 µm deep, composed of horizontally orientated, intricate and 3–5 μm wide hyphae. Hyphal terminations near the pileus margin unbranched, thin-walled, occasionally flexuous; terminal cells (12.5–)14.5–19.5–24.5(–26.0) × 4.5–5.0–5.5(–6.5) µm, mainly cylindrical, occasionally lageniform, apically mostly obtuse; subterminal cells usually shorter and slightly wider, ca. 4–7 µm wide, unbranched. Hyphal terminations near the pileus centre shorter and narrower than those near the pileus margin; terminal cells (11.0–)13.5–17.0–20.5(–21.5) × (3.5–)4.0–4.2–4.5 µm, clavate or cylindrical, apically obtuse; subterminal cells usually wider, ca. 4–6 µm, unbranched. ***Pileocystidia*** near the pileus margin always 1-celled, (20.0–)27.0–36.0–45.0(–48.0) × 4.5–5.5–6.0(–6.5) µm, clavate, occasionally fusiform, apically usually obtuse, sometimes with 2–6 µm long, round or elliptical appendage, thin-walled; contents crystalline, reddish-black in sulphovanillin. Pileocystidia near the pileus centre similar to those near the pileus margin, (20.0–)26.0–36.0–45.5(–52.5) × 4.0–5.0–5.5(–6.0) µm.

#### Habitat.

On the ground under mixed forests of *Pinusyunnanensis*, *Lithocarpusdealbatus* and *Quercus* spp.

#### Known distribution.

South-western China (Yunnan Province).

#### Additional specimens examined.

China, Yunnan Province, Chuxiong Yi Autonomous Prefecture, Lufeng City, Guangtong Town, 25°14'43"N, 101°45'53"E, 18 Sep 2022, X.L. Gao (RITF6329); ibid, 25°14'48"N, 101°45'28"E, alt. 2400 m, 24 Sep 2022, X.L. Gao (RITF6340); ibid, 25 Sep 2022, X.L. Gao (RITF6349); Kunming City, Wuhua District, Qiongzhu Temple, 25°3'58"N, 102°37'29"E, alt. 2150 m, 28 Jul 2012, Y.J. Hao (RITF3196).

#### Notes.

*Russulalavandula* is phylogenetically related to Thai *R.banwatchanensis* and Indian *R.pseudocyanoxantha* Paloi, K. Acharya & S. Khatua (Fig. 1). However, *R.lavandula* can be easily distinguished from them by its cracked pileus. Moreover, *R.banwatchanensis* differs in its darker coloured pileus, lack of lamellulae, thick-walled basidia and often longer pileocystidia of 42.5–127.0 × 2.5–5.0 µm (Crous et al. 2022) and *R.pseudocyanoxantha* differs in its darker coloured pileus, lack of lamellae furcations and association with *Shorearobusta* (Khatua et al. 2021). *Russulalavandula* can be easily confused with four Chinese species, via. *R.lotus*, *R.phloginea* J. Song & J.F. Liang, *R.purpureorosea* and *R.subpallidirosea* J.B. Zhang and L.H. Qiu in the field. However, *R.lotus*, originally described from southern China, can be easily distinguished by its absence of lamellae furcations, broadly ellipsoid to ellipsoid basidiospores, dispersed and wider hymenial cystidia on lamellae sides (52.0–70.0 × 10.0–16.0 μm) and longer cuticular terminal cells (10.0–40.0 × 4.0–8.0 μm) (Li and Deng 2018). *Russulaphloginea*, which occurs in subalpine areas, differs in having lamellae furcations only present near the stipe, smaller basidiospores of (6.0–)6.5–8.0 × 5.0–6.5 μm, longer hymenial cystidia of (48.0–)60.0–78.5(–79.5) × 7.5–9.5(–10.0) μm and pileocystidia with a moniliform apex (Song et al. 2019). *Russulapurpureorosea* and *R.subpallidirosea*, can be distinguished from *R.lavandula* by their rosy brown, pale pinkish-purple or pale greyish-pink pileus centre and occurrence at low altitudes (Zhang et al. 2017; Song 2022). Besides, *R.purpureorosea* lacks lamellae furcations and has shorter terminal cells of the pileipellis (6.5–15.5 × 2–5.5 μm) and wider pileocystidia (17.0–53.0 × 4.5–9.0 μm) (Song 2022). In addition, *R.cyanoxantha*, can be confused with *R.lavandula*. However, *R.cyanoxantha* can be distinguished by its uncracked pileus cuticle, longer hymenial cystidia up to 100 µm and slender cuticular hyphal end cells of 2–3 µm (Bon 1988; Sarnari 1998).

### ﻿ Russula lilaceofusca

Taxon classificationFungiRussulalesRussulaceae

Y.L. Chen & J.F. Liang, sp. nov.

B4B4A45E-28D5-58CF-BD41-D61BD9C9010A

851265

**Figure 8.**
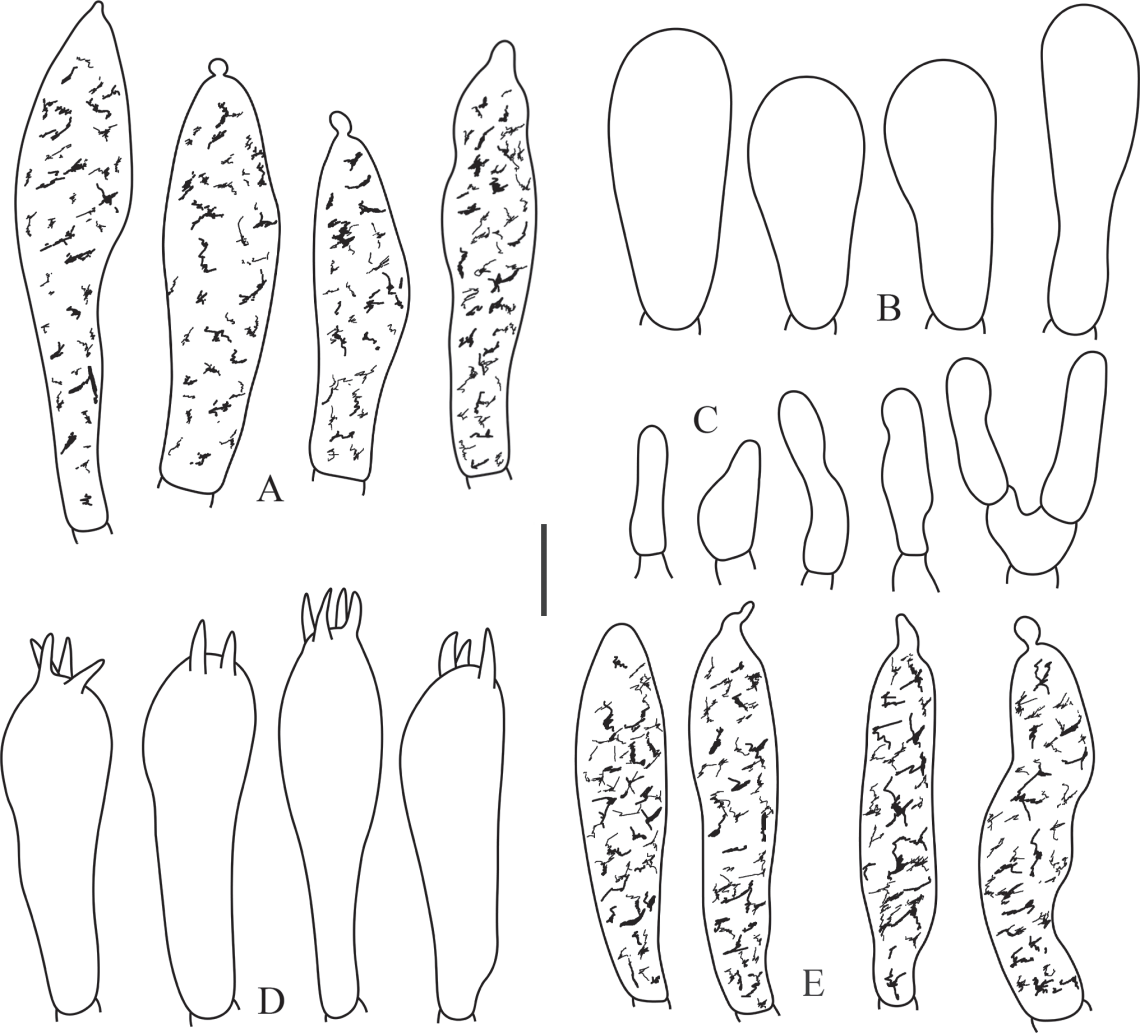
*Russulalilaceofusca* (RITF6330, holotype) **A** hymenial cystidia on lamellae sides **B** basidiola **C** marginal cells **D** basidia **E** hymenial cystidia on lamellae edges. Scale bar: 10 µm.

**Figure 9.**
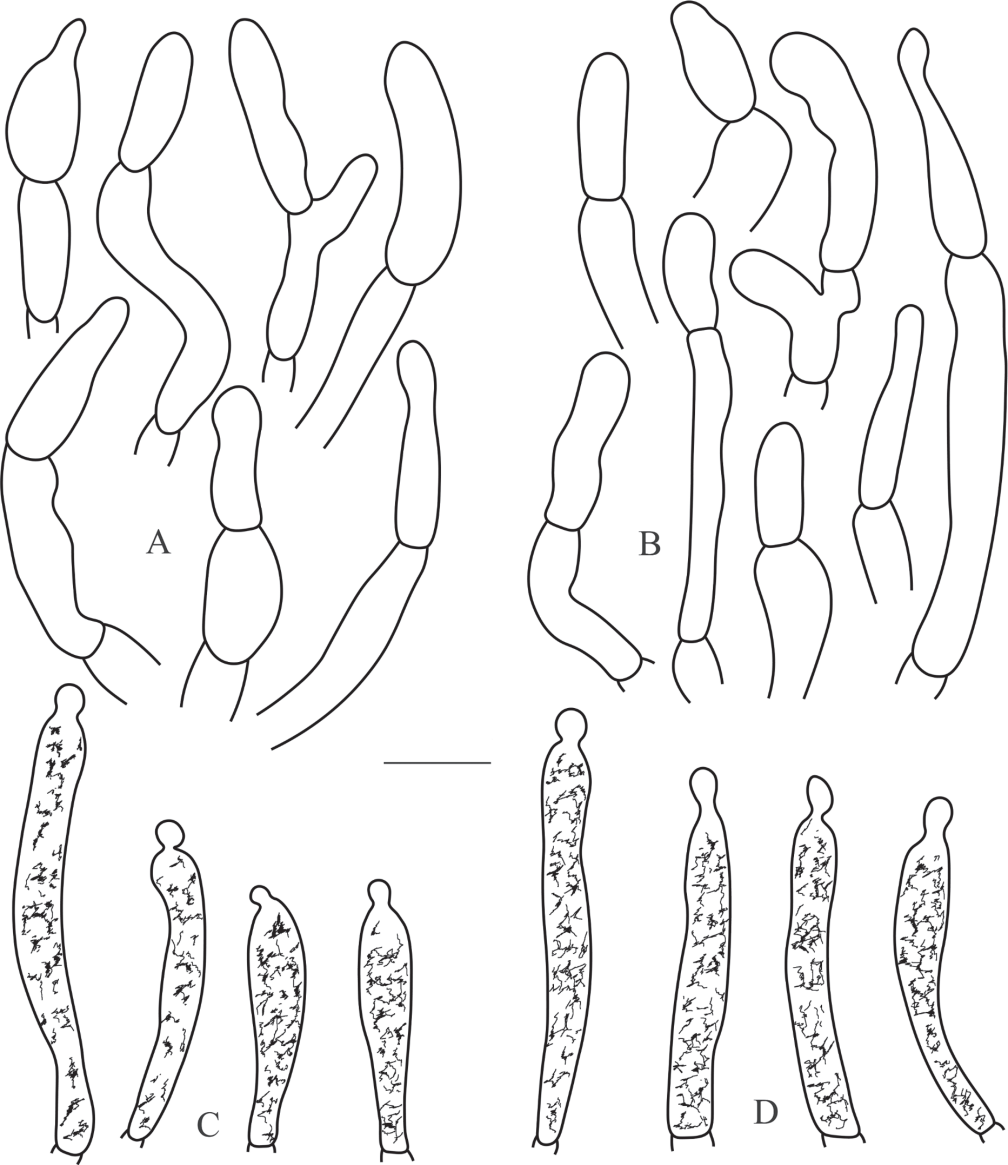
*Russulalilaceofusca* (RITF6330, holotype) **A** hyphal terminations near the pileus margin **B** hyphal terminations near the pileus centre **C** pileocystidia near the pileus margin **D** pileocystidia near the pileus centre. Scale bar: 10 µm.

Figs 2G–I , 3J–L , 8 , 9

#### Diagnosis.

*Russulalilaceofusca* is mainly characterised by its lilac brown to dark brown pileus, crowded lamellae with the presence of lamellulae and furcations, stipe often turning reddish-yellow when bruised, subreticulate basidiospores ornamentation and clavate hymenial cystidia often with capitate appendage whose contents change to reddish black in sulphovanillin. It can differ from *R.cyanoxantha* in shorter hymenial cystidia and wider cuticular terminal cells and differ from *R.fusiformata* in frequently present lamellulae and furcations, clavate hymenial cystidia on lamellae edges that have no reaction in sulphovanillin and occasionally branched cuticular hyphal terminations.

#### Holotype.

China, Yunnan Province, Chuxiong Yi Autonomous Prefecture, Lufeng City, G30).

#### Etymology.

‘*lilaceofusca*’ refers to a lilac brown pileus.

#### Description.

***Basidiomata*** medium-sized; pileus 40–60 mm in diameter, initially hemispherical when young, convex to applanate after mature; margin incurved, no striation, not cracked; surface dry, glabrous, reddish-white (8A2), lilac (15B4), brown (7E5) to dark brown (7F5). ***Lamellae*** adnate, very crowded, 20–24 per cm near pileus margin, cream, unchanging when bruised; lamellulae present and irregular in length; furcations frequently present; edge entire and concolorous. ***Stipe*** 35–50 × 8–17 mm, cylindrical to subcylindrical, slightly expanded towards the base, white (1A1), staining reddish-yellow (4A6) when touched, solid. ***Context*** white (1A1), unchanging when bruised, soft; taste mild; odour inconspicuous. ***Spore print*** not observed.

***Basidiospores*** (6.5–)7.0–7.8–8.7(–10.5) × (5.5–)6.1–6.9–7.7(–9.4) µm, Q = (1.05–)1.09–1.13–1.17(–1.26), subglobose to broadly ellipsoid; ornamentation of small to medium, dense (5–9 in a 3 μm diam. circle) amyloid warts, less than 0.5 μm high, subreticulate, connected by short line connections or ridges; suprahilar plage large, inamyloid. ***Basidia*** (26.5–)30.5–38.5–47.0(–56.0) × (9.5–)10.5–12.5–14.0(–15.5) µm, clavate or ellipsoid, 1- to 4-spored, thin-walled; basidiola clavate or ellipsoid, ca. 8.0–13.0 µm wide. ***Hymenial cystidia on lamellae sides*** dispersed to moderately numerous, (39.5–)43.5–48.5–53.5(–60.0) × (8.0–)9.5–10.5–11.5(–12.0) µm, mostly clavate, apically mostly obtuse, often with a 2.0–4.5 µm round appendage, thin-walled; contents granulose or heteromorphous, reddish-black in sulphovanillin. ***Hymenial cystidia on lamellae edges*** shorter and narrower than those on lamellae sides, (31.0–)36.0–41.5–47.0(–54.0) × (7.0–)7.5–8.5–10.0(–10.5) µm, mostly clavate, apically mostly obtuse, occasionally with a 2–3 µm long round or elliptical appendage, thin-walled; contents heteromorphous, reddish-black in sulphovanillin. ***Marginal cells*** (13.5–)15.0–18.5–22.0(–26.0) × 4.0–5.0–5.5(–6.5) µm, lageniform, clavate or subcylindrical. ***Pileipellis*** only hyphae of suprapellis metachromatic in cresyl blue, sharply delimited from the underlying context, two-layered, gelatinised; suprapellis 130–200 µm deep, composed of loosely arranged and erect hyphal terminations; subpellis 90–200 µm deep, composed of horizontally orientated and intricate hyphae. Hyphal terminations near the pileus margin occasionally branched, thin-walled, often flexuous; terminal cells (11.0–)12.5–18.5–24.0(–32.0) × (3.0–)3.5–4.0–5.0(–5.5) µm, mainly lageniform or cylindrical, apically obtuse, sometimes attenuated or constricted; subterminal cells usually longer and slightly wider, ca. 3.5–7.0 µm wide, occasionally branched. Hyphal terminations near the pileus centre slightly shorter than those near the pileus margin; terminal cells (9.5–)11.5–16.0–20.0(–25.5) × (3.0–)3.5–4.0–5.0(–5.5) µm, lageniform or cylindrical, apically obtuse, sometimes attenuated or constricted; subterminal cells usually equal or slightly wider, ca. 2.8–6.6 µm, occasionally branched. ***Pileocystidia*** near the pileus margin always 1-celled, (23.5–)26.5–35.0–43.5(–51.0) × (3.5–)4.0–4.5–5.5 µm, subfusiform or cylindrical, apically usually obtuse, always with 2–3 µm long, round or elliptical appendage, thin-walled; contents heteromorphous, reddish-black in sulphovanillin. Pileocystidia near the pileus centre similar to those near the pileus margin, (30.5–)34.5–39.5–44.5(–53.0) × 3.5–4.0–4.5(–5.5) µm.

#### Habitat.

On the ground of broad-leaved forests dominated by *Quercus* spp. or mixed forests with *Pinus* spp.

#### Known distribution.

Central (Hubei Province) and south-western China (Guizhou and Yunnan Provinces).

#### Additional specimens examined.

China, Guizhou Province, Zunyi City, Kuankuoshui National Nature Reserve, 28°14'29"N, 107°11'59"E, alt. 1750 m, 27 Sep 2014, H.J. Li (RITF3761); Hubei Province, Shennongjia Forest District, Jianglongping, 31°25'46"N, 110°20'18"E, alt. 1500 m, 9 Aug 2015, Y.K. Li (RITF2645); ibid, 11 Aug 2015, Y.K. Li (RITF2631).

#### Note.

Phylogenetic analyses showed that *R.lilaceofusca* was closely related to the Indian species *R.lakhanpalii* and *R.variata* from the United States. However, *R.lakhanpalii* differs in having a yellowish-white to pale yellow areolate pileus with orange-brown centre, cystidia that show no change in sulphovanillin and longer and wider pileocystidia of (27.0–)40.0–63.0–86.5(–123.0) × (4.0–)4.5–5.5–6.5(–7.0) μm near the pileus margin (Ghosh et al. 2020). *Russulavariata* differs in having a green pileus and basidiospores with higher warts of 0.4–1.0 µm (Hessler 1960; Burge 1979). In morphology, Chinese *R.fusiformata* Y. Song can be easily confused with this species. However, *R.fusiformata* can be distinguished by its striate pileus margin, absence of lamellulae and furcations, fusiform hymenial cystidia on the lamellae edges, cystidia that show a negative reaction in sulphovanillin and unbranched terminal cells in the pileipellis (Song 2022). *Russulalilaceofusca* is similar to *R.cyanoxantha*, but *R.cyanoxantha* has a greenish-violet pileus, longer hymenial cystidia up to 100 μm and slender cuticular hyphal end cells of 2–3 µm (Romagnesi 1967; Bon 1988; Sarnari 1998).

### ﻿ Russula perviridis

Taxon classificationFungiRussulalesRussulaceae

Y.L. Chen, B. Chen & J.F. Liang, sp. nov.

C9F61294-1D6D-5BD1-88F6-2B6BE7CA04A5

851266

**Figure 10.**
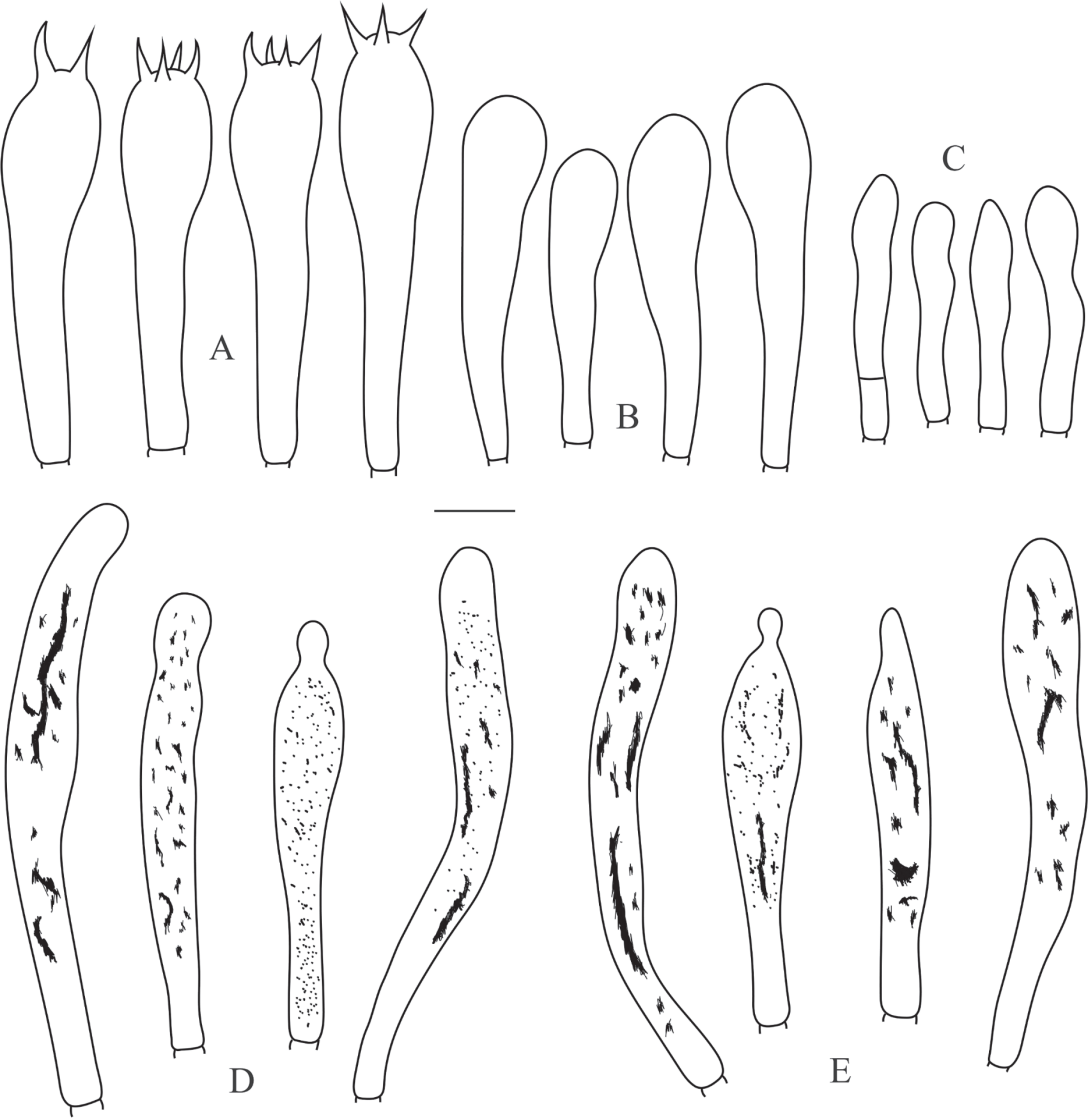
*Russulaperviridis* (RITF3131, holotype) **A** basidia **B** basidiola **C** marginal cells **D** hymenial cystidia on lamellae sides **E** hymenial cystidia on lamellae edges. Scale bar:10 µm.

**Figure 11.**
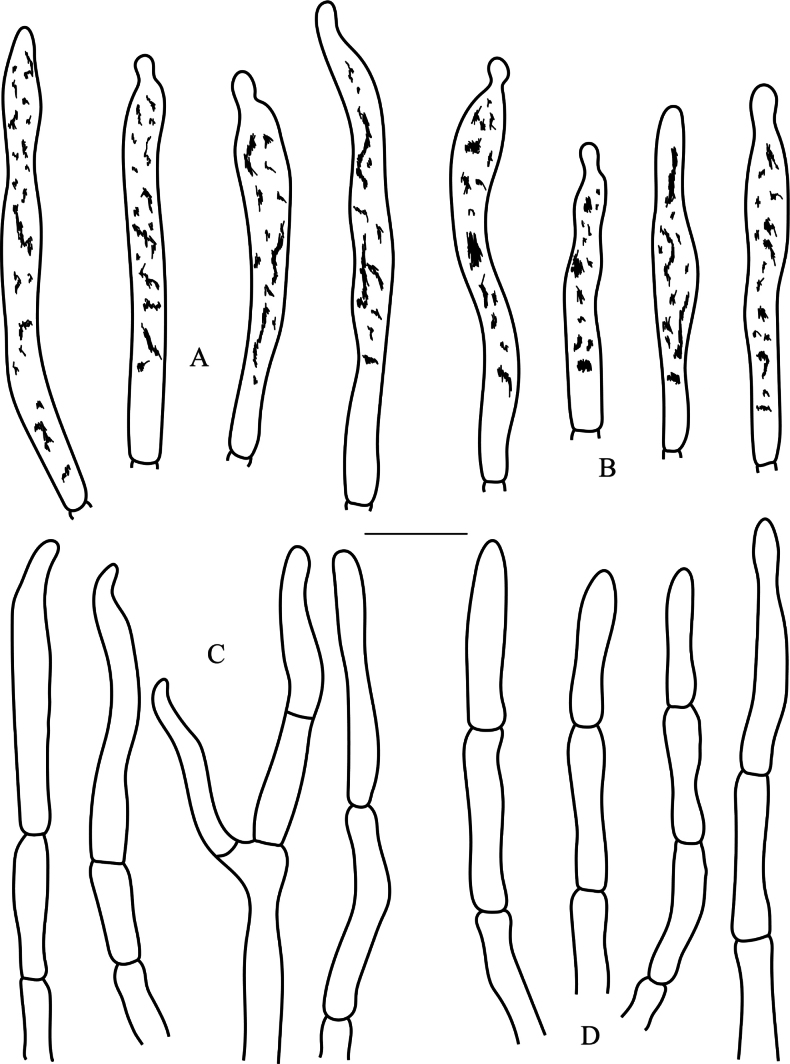
*Russulaperviridis* (RITF3131, holotype) **A** pileocystidia near the pileus margin **B** pileocystidia near the pileus centre **C** hyphal terminations near the pileus margin **D** hyphal terminations near the pileus centre. Scale bar: 10 µm.

Figs 2J–L , 3M–R , 10 , 11

#### Diagnosis.

*Russulaperviridis* is characterised by its large basidiomata, smooth pileus surface, frequently present lamellulae and furcations, a coarser stipe with yellow-brown tinge, globose to broadly ellipsoid basidiospores with locally reticulate ornamentation, long hymenial cystidia that turn greyish-black in sulphovanillin and is symbiotic with *Quercussemecarpifolia*. It differs from *R.dinghuensis* in longer and wider hymenial cystidia on lamellae edges, subreticulate basidiospores ornamentation and is associated with *Quercussemecarpifolia*. It differs from *R.nigrovirens* in frequently present furcations, subreticulate basidiospores ornamentation, longer and wider hymenial cystidia and related host plants.

#### Holotype.

China, Yunnan Province, Diqing Tibetan Autonomous Prefecture, Shangri La County, Bitahai Nature Reserve, 27°49'42"N, 99°59'30"E, alt. 3600 m, 20 Aug 2014, Q. Zhao (RITF3131).

#### Etymology.

‘*perviridis*’ refers to a dark green pileus.

#### Description.

***Basidiomata*** large-sized; pileus 90–150 mm in diameter, initially hemispherical to when young, applanate with a depressed centre when mature; margin usually incurved, striation short or inconspicuous, cracked when mature; surface dry, smooth and glabrous, peeling readily, greyish-green (27E5–30C5) to dark green (30F5), sometimes yellow-brownish (2C5) near the centre, greyish-yellow (2C3) or greenish-white (26A2) at the margin. ***Lamellae*** adnate to subfree, 9–12 per cm near pileus margin, white to cream, staining yellowish-brown (5E6) when bruised, 6–8 mm wide; lamellulae present and irregular in length; furcations frequently present near the stipe to half of the lamellae; edge entire and concolorous. ***Stipe*** 70–90 × 20–40 mm, cylindrical, white (1A1) with yellow-brown (2C5) tinge, solid. ***Context*** white (1A1), unchanging when bruised, 4–9 mm thick in half of the pileus radius, relatively soft; taste mild; odour inconspicuous. ***Spore print*** not observed.

***Basidiospores*** (5.9–)7.1–7.7–8.3(–9.7) × (5.2–)6.1–6.8–7.4(–8.4) µm, Q = (1.00–)1.07–1.14–1.21(–1.31), globose to broadly ellipsoid, hyaline in 5% KOH; ornamentation of small, dense (5–10 in a 3 μm diam. circle) amyloid warts, less than 0.5 μm high, reticulate, connected by short line connections or ridges; suprahilar plage large, inamyloid. ***Basidia*** (26.0–)34.5–42.0–59.0(–55.0) × (8.0–)9.5–11.0–12.0(–13.0) µm, clavate, 2- to 4-spored, thin-walled; basidiola clavate, ca. 6–11 µm wide. ***Hymenial cystidia on lamellae sides*** moderately numerous, (56.5–)62.5–69.5–77.0(–81.5) × (6.5–)7.0–7.5–8.0 µm, fusiform or clavate, apically mostly obtuse, sometimes acute, thin-walled; contents heteromorphous, greyish-black in sulphovanillin. ***Hymenial cystidia on lamellae edges*** similar to those on lamellae sides, (45.0)54.0–64.5–74.5(–79.5) × 6.0–7.5–8.5(–10.0) µm, mostly clavate, occasionally fusiform, apically mostly obtuse, sometimes with 3–5 µm long appendage, thin-walled; contents granulose or heteromorphous, greyish-black in sulphovanillin. ***Marginal cells*** (23.0–)24.5–27.5–33.5 × (4.5–)5.5–6.0–7.0 µm, clavate or subcylindrical, occasionally flexuous. ***Pileipellis*** only hyphae of suprapellis weakly metachromatic in cresyl blue, sharply delimited from the underlying context, 300–360 µm deep, two-layered, not gelatinised; suprapellis 180–200 µm deep, composed of erect and densely arranged hyphal terminations; subpellis 140–180 µm deep, composed of horizontally orientated, intricate and 3–5 μm wide hyphae. Hyphal terminations near the pileus margin occasionally branched, thin-walled, sometimes flexuous; terminal cells (17.0–)20.0–25.5–31.5(–38.0) × 3.0–4.0–4.5(–6.0) µm, usually clavate or subcylindrical, apically tapering; subterminal cells usually shorter, ca. 4–6 µm wide, occasionally branched. Hyphal terminations near the pileus centre shorter than those near the pileus margin; terminal cells (13.0–)14.5–20.5–26.5(–32.0) × 3.5–4.0–4.5 µm. ***Pileocystidia*** near the pileus margin always 1-celled, (36.5–)40.5–47.5–55.0(–60.0) × (3.0–)3.5–4.0–4.5 µm, fusiform or subcylindrical, apically usually obtuse, sometimes with 2–6 µm long, round or elliptical appendage, thin-walled; contents heteromorphous or granulose, greyish-black in sulphovanillin. Pileocystidia near the pileus centre similar to those near the pileus margin, (26.5–)28.0–36.0–44.0(–50.5) × 3.0–3.5–4.5 µm.

#### Habitat.

On the ground under broad-leaved forests dominated by *Quercussemecarpifolia*.

#### Known distribution.

South-western (Sichuan and Yunnan Provinces) and western China (Xizang Autonomous Region).

#### Additional specimens examined.

China, Sichuan Province, Ganzi Tibetan Autonomous Prefecture, Daocheng County, Yading Village, 4 Aug 2022, X.L. He (RITF6734); Yunnan Province, Diqing Tibetan Autonomous Prefecture, Deqin County, Wudi Lake, 27°48'47"N, 99°42'50"E, alt. 3300 m, 11 Sep 2023, Y.L. Chen & J.Y. Liang (RITF6982, 6983); Shangri La City, Bita Sea Scenic Area, 27°49'39"N, 99°59'28"E, alt. 3600 m, 15 Jul 2014, Q. Zhao (RITF3143); Xizang Autonomous Region, Linzhi City, Motuo County, 29°22'13"N, 95°26'59"E, alt. 1800 m, 15 Jul 2014, Q. Zhao (RITF2912); Lhasa City, Mozhugongka County, Zhaxigang Township, 29°42'10"N, 92°4'44"E, alt. 4500 m, 7 Aug 2023, Y. Zhang (RITF6790).

#### Notes.

Phylogenetic analyses showed that *R.perviridis* is related to two European species, *R.langei* Bon and *R.cyanoxantha* and the Chinese species *R.nigrovirens*. However, *R.langei* has a violaceous pileus, a white stipe with a lilac tinge and narrower cuticular hyphal terminations (2.0–3.0 μm) (Bon 1988), whereas *R.cyanoxantha* has non-reticulate basidiospore ornamentation and longer hymenial cystidia up to 100 μm (Bon 1988; Sarnari 1998). *Russulanigrovirens* differs in having patches on the pileus surface, furcations rarely present near the stipe, basidiospore ornamentation that does not form a reticulum and smaller hymenial cystidia (46.0–55.0 × 6.5–8.5 μm) on the lamellae edges (Zhao et al. 2015). Morphologically, *R.perviridis* has a green pileus as in Chinese *R.dinghuensis* J.B. Zhang & L.H. Qiu. However, *R.dinghuensis* differs in its shorter and narrower hymenial cystidia on lamellae edges (45.0–52.0 × 4.0–6.0 μm) and isolated basidiospore warts (Zhang et al. 2017). Furthermore, *R.perviridis* can be clearly distinguished from similar *Cyanoxanthinae* species in its habitat. *Russulaperviridis* is distributed in subalpine areas (over 2,000 m) and is associated with *Quercussemecarpifolia*. However, *R.nigrovirens* is associated with *Picea* spp., *Rhododendron* spp., *Sorbus* spp. and *Abies* spp. and *R.dinghuensis* is distributed in low-altitude areas (below 1,000 m).

##### ﻿Key to known species in R.subsect.Cyanoxanthinae in Asia

**Table:** 

1	Lamellulae absent or rare	**2**
–	Lamellulae irregularly inserted, but frequent (more than usual in subg. Heterophyllidiae)	**4**
2	Association with *Pinusmerkusii*	**3**
–	Association with Fagaceae	***R.fusiformata***
3	Lamellae and context changing pale purplish-pink when bruised, hymenial cystidia on lamellae edges numerous and short (35.0–57.5 × 10.0–10.5 μm)	***R.lilacina***
–	Lamellae and context unchanging when bruised, hymenial cystidia on lamellae edges dispersed to moderately numerous and long (45.0–77.5 × 7.5–10.0 μm)	***R.purpureoviridis***
4	Basidiospores ornamentation mainly subreticulate to reticulate	**5**
–	Basidiospores ornamentation mainly isolated	**7**
5	Lamellae instant (9–12/cm at pileus margin), average length of hymenial cystidia on lamellae edges over 50 μm	***R.perviridis***
–	Lamellae crowed to very crowed (14–27/cm at pileus margin), average length of hymenial cystidia on lamellae edges less than 50 μm	**6**
6	Hymenial cystidia on lamellae sites short and wide of (39.5–)43.5–48.5–53.5(–60.0) × (8.0–)9.5–10.5–11.5(–12.0) µm, clavate, pileocystidia near the pileus margin short and narrow [(23.5–)26.5–35.0–43.5(–51.0) × (3.5–)4.0–4.5–5.5] µm	***R.lilaceofusca***
–	Hymenial cystidia on lamellae sites short and wide of (45–)47.2–548.5–61.8(–75) × (6–)6.6–7.3–7.9(–8) µm, cylindrical to subclavate to fusiform, pileocystidia near the pileus margin long and wide [(27–)39.8–63.1–86.5(–123) × (4–)4.4–5.4–6.4(–7)] µm	***R.lakhanpalii***
7	Lamellae furcations absent or rare	**8**
–	Lamellae furcations irregularly inserted, but frequent (more than usual in subg. Heterophyllidiae)	**12**
8	Appearing in subalpine forest dominated by *Picea* spp., *Rhododendron* spp., *Sorbus* spp. and *Abies* spp.	***R.nigrovirens***
–	Appearing in evergreen broad-leaved forest dominated by *Shorearobusta*, *Lithocarpuscorneus*, *Quercus* spp.	**9**
9	Hymenial cystidia on lamellae edges narrow (not exceeding 7 µm wide)	***R.dinghuensis***
–	Hymenial cystidia on lamellae edges wide (most cells exceeding 7 µm wide)	**9**
10	Basidiospores broadly ellipsoid to ellipsoid, occasionally subglobose	***R.lotus***
–	Basidiospores subglobose to broadly ellipsoid	**11**
11	Lamellae adnate, context unchanging when bruising or with FeSO_4_, hymenial cystidia on lamellae edges short (36–64.5 × 7.2–10.8 µm), pileocystidia short and narrow (18.2–32 × 3.5–5.4 µm)	***R.pseudocyanoxantha***
–	Lamellae adnexed to subdecurrent, context changing light yellow when bruising or with FeSO_4_, hymenial cystidia on lamellae edges long (54–60–95 × 7–8.5–10 µm), pileocystidia short and narrow (17–53 × 4.5–9 µm)	***R.purpureorosea***
12	Basidiospores globose to subglobose (Q < 1.15)	***R.banwatchanensis***
–	Basidiospores subglobose to ellipsoid (Q = 1.15–1.45)	**13**
13	Basidiospores broadly ellipsoid to ellipsoid (Q = 1.30–1.45)	***R.subpallidirosea***
–	Basidiospores subglobose to broadly ellipsoid (Q = 1.15–1.30)	**14**
14	Pileocystidia often with moniliform appendage	***R.phloginea***
–	Pileocystidia with capitate appendage	**15**
15	Pileal margin tuberculate-striate, lamellae distant (5–8/cm near the pileus margin), hymenial cystidia on lamellae edges long and wide [(54.5–)58.0–65.0–71.5(–75.5) × (8.0–)9.0–10.5–11.5(–13.0)] µm, fusiform, cuticular hyphal terminations branched	***R.atrochermesina***
–	Pileal margin indistinctly or not striate, lamellae slightly crowded (10–13/cm near the pileus margin), hymenial cystidia on lamellae edges short and narrow [(35.0–)39.1–45.8–52.5(–56.0) × (6.0–)7.0–8.1–9.2(–10.0)] µm, mostly clavate or subcylindrical, cuticular hyphal terminations unbranched	***R.lavandula***

## ﻿Discussion

Our multi-gene phylogenetic analyses (Fig. 1) indicate that species with white spore print, basidiospores ornamented by warts with inamyloid suprahilar spot, metachromatic pileipellis and one-celled pileocystidia constitute Cyanoxanthinae. The analyses also suggest that *Cyanoxanthinae* can be divided into two major lineages, which may be distinguishable by the connections of basidiospores ornamentation. Clade 1 represented by *R.lilaceofusca* and *R.perviridis* has more connections between the warts. Clade 2 represented by *R.lavandula* and *R.atrochermesina* has more isolated warts. Additional studies are need to determine the critical point for the number or density of connections. There are no other morphological differences that can clearly correspond to these two lineages.

We recommend several main characteristics that can be used to distinguish species within *Cyanoxanthinae*. The first, lamellulae and furcations are crucial characters in our view. Except for *R.fusiformata* having completely regular lamellae, the remaining species in *Cyanoxanthinae* have inserted lamellulae or furcations. The density of lamellulae and furcations may require our attention in future research. The second, the species of *Cyanoxanthinae* have different shape of basidiospores (Q values). Four Chinese species, *R.dinghuensis*, *R.nigrovirens*, *R.lotus* and *R.subpallidirosea* have ellipsoid basidiospores (Q > 1.3), while other species have broadly ellipsoid basidiospores (Q < 1.3).

The Cyanoxanthinae is a widely distributed group, ranging from tropical forests to temperate regions and have formed ectomycorrhizal relationships with various host plants. All known species come from the Northern Hemisphere, except for one species from Madagascar. Australia is an unexplored continent for studying this group. For the level of species, there seems to be restricted life zones and specific hosts. For example, we found that *R.perviridis* is only distributed in the subalpine zone and only forms a symbiotic relationship with *Quercussemecarpifolia*. The European *R.cyanoxantha* is well-known to many people in China. Despite our team conducting surveys in the southern provinces of China (subtropical regions) for over a decade, we have not found it there. This means it is a strictly temperate species.

Currently, it is known that there are 16 species in the Cyanoxanthinae distributed in Asia, including the four new species described in this article. These species are all found from tropical or subtropical regions and, so far, there have been no new species found from temperate Asia. In view of the wide distribution of members of the subsection in Europe, more attention needs to be paid to temperate Asia.

Amongst the 12 known species in China, we can only confirm that *R.phloginea* is edible and more support is needed for the edibility of the remaining species.

## Supplementary Material

XML Treatment for Russula atrochermesina

XML Treatment for Russula lavandula

XML Treatment for Russula lilaceofusca

XML Treatment for Russula perviridis

## ﻿Additional information

### Author ORCIDs

Yanliu Chen https://orcid.org/0000-0002-8054-2517

Bin Chen https://orcid.org/0000-0001-8459-3752

Shengkun Wang https://orcid.org/0000-0002-2487-6344

Mengya An https://orcid.org/0009-0001-6170-5886

Jinhua Zhang https://orcid.org/0000-0001-8523-644X

Jingying Liang https://orcid.org/0000-0003-4111-4859

Yaxin Wang https://orcid.org/0009-0002-1302-5154

Xuelian Gao https://orcid.org/0009-0006-6059-4877

Junfeng Liang https://orcid.org/0000-0002-0433-2577

### Data availability

All of the data that support the findings of this study are available in the main text or Supplementary Information.

## Supplementary materials

Supplementary material 1
ITS
Data type fas https://binary.pensoft.net/file/1089912This dataset is made available under the Open Database License (http://opendatacommons.org/licenses/odbl/1.0/). The Open Database License (ODbL) is a license agreement intended to allow users to freely share, modify, and use this Dataset while maintaining this same freedom for others, provided that the original source and author(s) are credited.Yanliu Chen, Bin Chen, Ruoxi Liang, Shengkun Wang, Mengya An, Jinhua Zhang, Jingying Liang, Yaxin Wang, Xuelian Gao, Junfeng Liang

Supplementary material 2 LSU Data type fas https://binary.pensoft.net/file/1089913This dataset is made available under the Open Database License (http://opendatacommons.org/licenses/odbl/1.0/). The Open Database License (ODbL) is a license agreement intended to allow users to freely share, modify, and use this Dataset while maintaining this same freedom for others, provided that the original source and author(s) are credited.Yanliu Chen, Bin Chen, Ruoxi Liang, Shengkun Wang, Mengya An, Jinhua Zhang, Jingying Liang, Yaxin Wang, Xuelian Gao, Junfeng Liang

Supplementary material 3 RPB2 Data type fas https://binary.pensoft.net/file/1089914This dataset is made available under the Open Database License (http://opendatacommons.org/licenses/odbl/1.0/). The Open Database License (ODbL) is a license agreement intended to allow users to freely share, modify, and use this Dataset while maintaining this same freedom for others, provided that the original source and author(s) are credited.Yanliu Chen, Bin Chen, Ruoxi Liang, Shengkun Wang, Mengya An, Jinhua Zhang, Jingying Liang, Yaxin Wang, Xuelian Gao, Junfeng Liang

Supplementary material 4 SSU Data type fas https://binary.pensoft.net/file/1089915This dataset is made available under the Open Database License (http://opendatacommons.org/licenses/odbl/1.0/). The Open Database License (ODbL) is a license agreement intended to allow users to freely share, modify, and use this Dataset while maintaining this same freedom for others, provided that the original source and author(s) are credited.Yanliu Chen, Bin Chen, Ruoxi Liang, Shengkun Wang, Mengya An, Jinhua Zhang, Jingying Liang, Yaxin Wang, Xuelian Gao, Junfeng Liang
